# Metabolic liver burden and osteoarthritis prevalence: A comparative analysis of noninvasive hepatic indices

**DOI:** 10.1097/MD.0000000000048764

**Published:** 2026-05-22

**Authors:** Di Wang, Xin Zhao

**Affiliations:** a Department of Orthopedics, The First Hospital of China Medical University, Shenyang, Liaoning Province, China; b Department of Geratology, The First Hospital of China Medical University, Shenyang, Liaoning Province, China.

**Keywords:** Fibrosis-4 index, hepatic steatosis index, liver fibrosis, metabolic phenotype, metabolic syndrome, NAFLD fibrosis score, osteoarthritis

## Abstract

Osteoarthritis (OA) is increasingly recognized as a condition with a distinct metabolic phenotype, characterized by systemic low-grade inflammation and metabolic dysregulation. Concurrent with the rising prevalence of OA is the global burden of metabolic dysfunction-associated steatotic liver disease. This study aimed to evaluate and compare the associations of 3 noninvasive liver scores – hepatic steatosis index (HSI), NAFLD fibrosis score (NFS), and Fibrosis-4 index (FIB-4) – with OA prevalence among US adults. We performed a cross-sectional analysis of National Health and Nutrition Examination Survey cycles (1999–2018). HSI, NFS, and FIB-4 were calculated and examined both as quartiles and per standard deviation increment. Associations with self-reported OA were estimated using survey-weighted modified Poisson regression with robust variance (prevalence ratios) as the primary approach, with survey-weighted logistic regression (odds ratios) performed as a secondary analysis. The final sample included 40,447 participants. In fully adjusted Poisson regression models (Model 3), HSI demonstrated the most robust and consistent association with OA. Participants in the highest HSI quartile exhibited a 2.09-fold higher OA prevalence compared with the lowest quartile, with a dose–response relationship. NFS demonstrated a moderate association, though substantially attenuated compared with its crude estimate after adjustment for demographic, socioeconomic, lifestyle, and non-embedded clinical covariates. Notably, the association between FIB-4 and OA was largely confounded by age; after adjustment, FIB-4 showed only a modest association in the highest quartile, whereas the continuous association was null. Sensitivity analyses consistently showed that HSI had the strongest and most stable association with OA among the 3 indices. The steatosis-oriented metabolic burden reflected by HSI shows a stronger adjusted association with OA than the more age-dependent fibrosis marker FIB-4 in the general population. These results support the view that steatosis-related metabolic dysfunction may be more relevant to OA burden than hepatic fibrotic burden alone. Because HSI incorporates body mass index and diabetes status, the observed association should be interpreted as that of a composite metabolic marker rather than a fully independent effect of hepatic steatosis itself. HSI may therefore be useful as a simple indicator of a steatosis-oriented metabolic OA profile in integrated risk assessment.

**Key Points**

- Robust association of HSI with OA: HSI showed the strongest and most consistent adjusted association with OA prevalence compared with the more fibrosis-oriented scores NFS and FIB-4 under the predefined score-specific adjustment strategy.
- Metabolic phenotype context: The stronger associations observed for HSI and NFS, both of which incorporate metabolic parameters such as BMI and diabetes, are consistent with a steatosis-oriented metabolic OA phenotype, although part of these associations may reflect the contribution of embedded metabolic risk factors.
- Age confounding in FIB-4: FIB-4 showed limited utility for OA risk assessment in the general population, with its crude association being largely explained by age confounding rather than an independent metabolic or fibrotic signal, cautioning against its isolated use in this context.
- Large-scale evidence: Utilizing a large, nationally representative sample from NHANES (1999–2018) provides high statistical power and generalizability, strengthening population-level evidence for an association between liver-related metabolic indices and OA.
- Clinical utility: HSI may function as a simple composite marker of a steatosis-oriented metabolic OA profile, supporting integrated evaluation of metabolic and musculoskeletal health rather than a liver-specific diagnostic interpretation.

## 1. Introduction

Osteoarthritis (OA) is a leading cause of pain, disability, and work loss worldwide, imposing a growing societal and healthcare burden.^[1,2]^ While age, trauma, and biomechanical stress remain important determinants, accumulating evidence has redefined OA from a purely mechanical “wear-and-tear” disorder to a heterogeneous whole-joint disease encompassing multiple pathophysiological phenotypes.^[3–5]^ Among these, the metabolic phenotype has attracted increasing attention: OA can affect both weight-bearing and non–weight-bearing joints and has been increasingly linked to obesity-related adipose dysfunction and chronic low-grade metabolic inflammation, supporting the concept that systemic metabolic drivers contribute to OA beyond mechanical loading alone.^[6,7]^

In parallel, metabolic dysfunction-associated steatotic liver disease (MASLD; formerly nonalcoholic fatty liver disease [NAFLD]) has emerged as one of the most common chronic liver conditions globally and is increasingly regarded as the hepatic manifestation of systemic metabolic dysfunction.^[8,9]^ MASLD spans a continuum from steatosis to metabolic dysfunction-associated steatohepatitis and progressive fibrosis.^[10]^ Beyond serving as a metabolic target organ, the liver functions as a central endocrine and immunometabolic hub that orchestrates systemic lipid-glucose homeostasis and modulates inflammatory signaling cascades.^[11]^ This raises the possibility of liver-joint crosstalk, whereby hepatic metabolic dysfunction and liver-derived mediators may contribute to extrahepatic joint pathology through systemic inflammatory and metabolic perturbations relevant to the metabolic OA phenotype.^[12,13]^

Because liver biopsy is unsuitable for population research and routine screening, musculoskeletal epidemiology necessarily relies on noninvasive scores derived from routine anthropometric and laboratory measures.^[14–16]^ Three widely used indices capture partially distinct domains of liver-related metabolic injury.^[17]^ The hepatic steatosis index (HSI) was developed as a simple screening tool for hepatic steatosis, incorporating body mass index (BMI), diabetes status, and the alanine aminotransferase (ALT)/aspartate aminotransferase (AST) ratio to capture metabolic burden directly.^[18,19]^ The NAFLD fibrosis score (NFS) was developed to stratify advanced fibrosis risk in NAFLD cohorts and integrates age, BMI, impaired fasting glucose/diabetes, platelet count, albumin, and the AST/ALT ratio – thus capturing both metabolic burden and indicators of hepatic synthetic function and fibrotic tendency.^[20,21]^ The Fibrosis-4 index (FIB-4), originally proposed in viral hepatitis and widely adopted for ruling out advanced fibrosis, relies on age, AST, ALT, and platelet count.^[22]^ Because age is a direct component of the FIB-4 formula, this index may be disproportionately influenced by chronological aging, potentially reducing its specificity in older populations and limiting its ability to capture metabolic dysfunction when applied to extrahepatic outcomes such as OA.^[23]^

Despite growing interest in metabolic OA and MASLD, epidemiologic evidence linking fatty liver disease to OA remains limited and methodologically heterogeneous. In a Korean national cross-sectional study, MAFLD defined by the fatty liver index was associated with a 1.48-fold higher probability of radiographic knee OA.^[24]^ In a US National Health and Nutrition Examination Survey (NHANES) 2017 to 2018 analysis, vibration-controlled transient elastography-defined NAFLD was independently associated with self-reported OA.^[25]^ More recently, a large UK Biobank prospective cohort reported that MAFLD was associated with a higher risk of incident knee OA, with risk increasing according to fibrosis severity.^[26]^ Collectively, however, prior studies have focused on disease definitions or selected liver-related metrics rather than directly comparing commonly used noninvasive liver scores such as HSI, NFS, and FIB-4 within a unified analytic framework. Such a head-to-head comparison is clinically informative: if indices that capture metabolic burden (HSI and NFS) show stronger and more robust associations with OA than a predominantly age-driven fibrosis marker (FIB-4), this would suggest that steatosis-related metabolic dysfunction may be more relevant to OA burden than hepatic fibrotic scarring alone in the general population. To address this gap, we used data from a large, nationally representative sample of US adults to conduct a head-to-head comparative analysis of HSI, NFS, and FIB-4 in relation to OA prevalence. We hypothesized that HSI and NFS would show stronger adjusted associations with OA than FIB-4 under the predefined score-specific adjustment strategy.

## 2. Methods

### 2.1. Study design and data source

We conducted a cross-sectional analysis using data from the NHANES, an ongoing program of the National Center for Health Statistics (NCHS), Centers for Disease Control and Prevention.^[27,28]^ NHANES uses a complex, multistage, stratified, clustered probability sampling design to assess the health and nutritional status of the civilian, noninstitutionalized US population. The NCHS Research Ethics Review Board approved all NHANES protocols, and written informed consent was obtained from all participants.

### 2.2. Study population

We pooled data from 10 consecutive 2-year NHANES cycles (1999–2000 through 2017–2018) to maximize statistical power and enable robust subgroup analyses. Our study population was restricted to adults aged 20 years and older who completed the interview and underwent examination at the mobile examination center (MEC), because OA ascertainment in NHANES was based on arthritis-related questionnaire items administered only to participants aged ≥20 years across the included survey cycles. We sequentially excluded participants with missing or indeterminate OA status and those without sufficient information to calculate any of the 3 liver indices. Participants with at least 1 calculable liver score (HSI, NFS, or FIB-4) were retained in the common analytic cohort. Because component availability differed across indices, score-specific quartile analyses were conducted among participants with non-missing values for the corresponding score.

### 2.3. Exposure assessment: noninvasive liver scores

Three established noninvasive liver indices were calculated using their original published formulas. HSI was calculated as 8 × (ALT/AST ratio) + BMI (+2 if female; +2 if diabetes), as originally proposed by Lee et al for steatosis screening.^[29]^ NFS was calculated as −1.675 + 0.037 × age (years) + 0.094 × BMI (kg/m^2^) + 1.13 × impaired fasting glucose/diabetes (yes = 1, no = 0) + 0.99 × AST/ALT ratio − 0.013 × platelet count (×10^9^/L) − 0.66 × albumin (g/dL), according to the original model developed by Angulo et al.^[30]^ FIB-4 was calculated as age (years) × AST (U/L)/(platelet count [10^9^/L] × √ALT [U/L]), consistent with the original derivation by Sterling et al.^[31]^

Each liver score was analyzed both continuously (per 1-standard deviation [SD] increment) and categorically (quartiles Q1–Q4) to capture dose–response relationships and identify potential nonlinear or threshold effects.

### 2.4. Outcome assessment: OA

The primary outcome was the presence of OA, defined based on the Medical Conditions Questionnaire. Participants responding affirmatively to “Has a doctor or other health professional ever told you that you had arthritis?” were subsequently asked to specify the type of arthritis. Participants were classified as having OA if they specifically reported “OA” or “Degenerative arthritis” as the diagnosis.

### 2.5. Covariates and model specification

Covariates were selected a priori based on biological plausibility and prior literature. Demographic variables included age, sex, and race/ethnicity (non-Hispanic White, non-Hispanic Black, Mexican American, Other Hispanic, and Other Race). Socioeconomic and lifestyle covariates included education level, poverty income ratio, marital status, and alcohol consumption status. Comorbidities included hypertension, diabetes, and a history of cardiovascular disease (CVD). Hypertension was defined as measured elevated blood pressure (mean systolic ≥140 mm Hg and/or mean diastolic ≥90 mm Hg), self-reported physician diagnosis, or current use of antihypertensive medications. Diabetes was defined by self-reported physician diagnosis, and CVD history was defined as a composite of self-reported congestive heart failure, coronary heart disease, angina, myocardial infarction, or stroke.

To reduce overadjustment, mathematical coupling, and multicollinearity, Model 3 used a score-specific adaptive adjustment strategy rather than an identical covariate set for all liver indices. For HSI and NFS, BMI and diabetes/impaired fasting glucose were not additionally entered into Model 3 because these variables are explicit components of the original score formulas. Accordingly, the fully adjusted models for HSI and NFS should be interpreted as adjusted for major demographic, socioeconomic, lifestyle, and clinical confounders other than those already embedded in the score algorithms. In contrast, for FIB-4, BMI and diabetes were additionally included in Model 3 because FIB-4 does not explicitly incorporate adiposity or glycemic status.

### 2.6. Statistical analysis

All analyses accounted for the NHANES complex survey design (strata, primary sampling units, and sampling weights). For the pooled 1999 to 2018 analysis, we created combined MEC examination weights by dividing the 2-year MEC weights (WTMEC2YR) by the number of included cycles (10), consistent with NCHS guidance for combining multiple cycles.

Baseline characteristics are presented as survey-weighted means (with SDs) for continuous variables and weighted percentages for categorical variables. Between-quartile differences were evaluated using the design-based Kruskal–Wallis test for continuous variables and the Rao–Scott chi-square test for categorical variables.

Given that OA is a relatively common outcome (weighted prevalence >10%), we selected survey-weighted Poisson regression with robust variance (modified Poisson approach) as the primary analytic method to estimate prevalence ratios (PRs), which avoid overestimation of effect sizes inherent to odds ratios (ORs) when outcomes are common. Survey-weighted logistic regression was additionally performed to obtain ORs and is reported in the Supplemental Digital Content for comparison. Linear trends across quartiles were evaluated by modeling the median value of each quartile (weighted by the survey design) as a continuous variable in the regression models.

Dose–response shape and potential nonlinearity were assessed using restricted cubic splines with 4 knots placed at the 5th, 35th, 65th, and 95th percentiles, with the weighted median as the reference; nonlinearity was tested using Wald tests. Effect modification was explored through stratified analyses and interaction terms, with design-adjusted Wald tests. Multiple interaction tests were controlled using the Benjamini–Hochberg false discovery rate (BH-FDR) procedure.

Multiple sensitivity analyses were performed to assess robustness: excluding underweight participants (BMI < 18.5 kg/m^2^); excluding heavy alcohol consumers (sex-specific thresholds); excluding individuals with markedly elevated transaminases (ALT or AST >120 U/L) to minimize potential acute hepatocellular injury; excluding participants with diabetes and/or CVD; and calculating *E*-values to quantify the minimum strength of unmeasured confounding needed to nullify observed associations. All tests were two-sided, with statistical significance defined as *P* < .05. Effect estimates and 95% confidence intervals (CIs) are reported to 2 decimal places, and exact *P* values are reported to 3 decimal places unless *P* < .001. All statistical analyses were performed using R version 4.5.1 (R Foundation for Statistical Computing).

## 3. Results

### 3.1. Baseline characteristics of the study population

**Figure 1.**
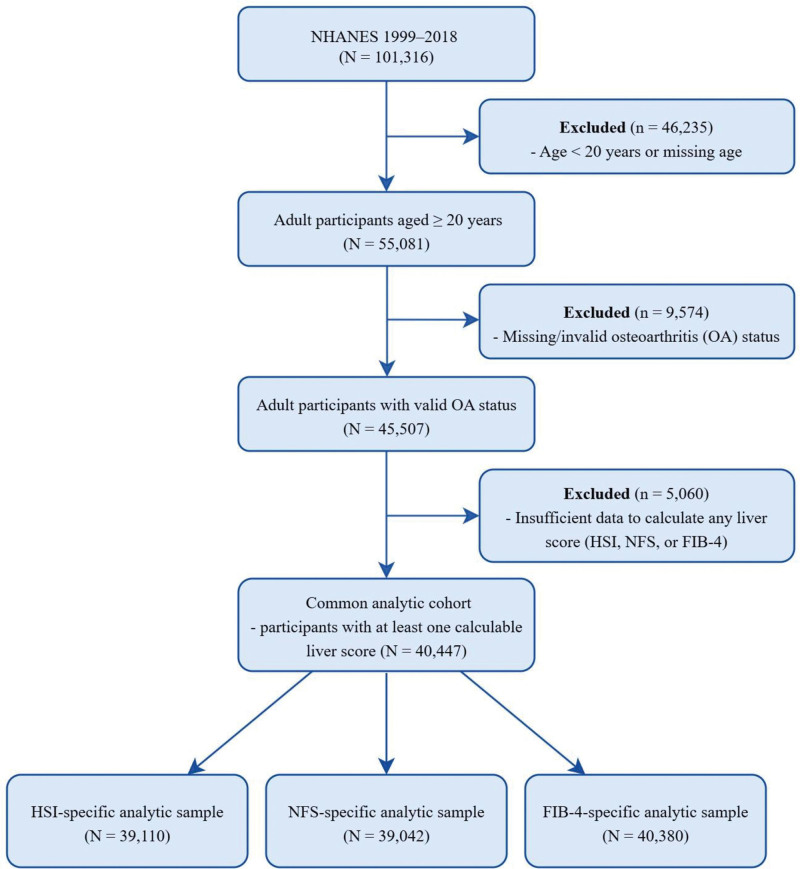
Flowchart of participant selection from NHANES 1999 to 2018 and derivation of the final analytic sample. FIB-4 = Fibrosis-4 index, HSI = hepatic steatosis index, NFS = NAFLD fibrosis score, NHANES = National Health and Nutrition Examination Survey, OA = osteoarthritis.

Figure 1 summarizes the study sample selection and the derivation of the common analytic cohort. Of 101,316 NHANES participants (1999–2018), we sequentially excluded 46,235 individuals aged <20 years or with missing age data and 9574 with missing or invalid OA status. We then retained 40,447 adults in the common analytic cohort, defined as participants with at least 1 calculable liver index (HSI, NFS, or FIB-4). Because the 3 indices required different component variables, score-specific analytic samples differed slightly: 39,110 for HSI, 39,042 for NFS, and 40,380 for FIB-4. Thus, the main analysis used a unified adult cohort with only minimal score-specific variation in sample size. Weighted baseline characteristics across quartiles of HSI are presented in Table 1. Baseline characteristics of the common analytic cohort are shown in Table S1, and the corresponding quartile-based distributions for NFS and FIB-4 are shown in Tables S2 and S3.

**Table 1:** Characteristics by HSI quartiles (weighted).

Characteristic	Overall‡	Q1	Q2	Q3	Q4	*P* value†
	N = 39,110*	N = 9778*	N = 9777*	N = 9777*	N = 9778*	
Age	44.86 (16.48)	42.24 (17.68)	46.29 (16.93)	46.74 (15.89)	44.33 (14.81)	<.001
Sex						<.001
Male	19,187 (49%)	4994 (46%)	5065 (51%)	5000 (53%)	4128 (45%)	
Female	19,923 (51%)	4784 (54%)	4712 (49%)	4777 (47%)	5650 (55%)	
Race						<.001
Non-Hispanic White	17,132 (68%)	4691 (71%)	4564 (70%)	4022 (66%)	3855 (64%)	
Non-Hispanic Black	7586 (10%)	1862 (9.8%)	1649 (8.9%)	1813 (10%)	2262 (13%)	
Hispanic	10,497 (15%)	1753 (10.0%)	2501 (13%)	3153 (18%)	3090 (18%)	
Other	3895 (7.2%)	1472 (9.7%)	1063 (7.6%)	789 (6.2%)	571 (5.1%)	
Education						<.001
>High school	20,440 (61%)	5500 (65%)	5181 (62%)	4845 (59%)	4914 (58%)	
High school	8865 (23%)	2086 (21%)	2204 (23%)	2237 (24%)	2338 (26%)	
<High school	9767 (16%)	2179 (14%)	2382 (15%)	2688 (17%)	2518 (16%)	
PIR	3.05 (1.64)	3.03 (1.67)	3.19 (1.62)	3.08 (1.63)	2.90 (1.62)	<.001
Marital						<.001
Married/living with partner	23,878 (64%)	5392 (58%)	6201 (66%)	6268 (68%)	6017 (65%)	
Not married	14,855 (36%)	4306 (42%)	3472 (34%)	3407 (32%)	3670 (35%)	
BMI	28.44 (6.55)	21.90 (2.16)	25.90 (1.90)	29.44 (2.30)	36.87 (6.02)	<.001
Drinking status						<.001
Never	6818 (15%)	1583 (14%)	1680 (15%)	1686 (15%)	1869 (17%)	
Former	3891 (9.1%)	806 (6.9%)	966 (9.0%)	1040 (9.8%)	1079 (11%)	
Current	24,709 (76%)	6407 (79%)	6208 (76%)	6126 (75%)	5968 (72%)	
Diabetes	3913 (7.4%)	249 (1.5%)	657 (4.3%)	1158 (8.4%)	1849 (16%)	<.001
Hypertension	11,450 (26%)	1754 (14%)	2567 (23%)	3185 (30%)	3944 (39%)	<.001
CVD_history	3196 (6.5%)	672 (4.8%)	823 (6.5%)	838 (7.0%)	863 (7.9%)	<.001
OA_case	4386 (12%)	742 (7.5%)	1054 (11%)	1166 (13%)	1424 (16%)	<.001
ALT	25.54 (22.70)	18.93 (16.42)	22.92 (17.61)	26.74 (14.55)	33.97 (33.92)	<.001
AST	25.09 (16.21)	24.69 (19.85)	24.76 (17.94)	24.75 (10.88)	26.16 (14.27)	<.001
ALB	4.29 (0.35)	4.39 (0.34)	4.32 (0.34)	4.28 (0.33)	4.17 (0.34)	<.001
PLT	253.63 (64.75)	246.64 (62.85)	249.11 (62.79)	254.06 (63.64)	265.15 (68.11)	<.001
HSI	37.60 (7.77)	29.22 (2.20)	34.37 (1.26)	39.06 (1.53)	48.20 (6.00)	<.001
NFS	−2.30 (1.42)	−2.79 (1.26)	−2.45 (1.31)	−2.22 (1.35)	−1.71 (1.51)	<.001
FIB-4	1.00 (0.76)	1.07 (0.89)	1.06 (0.72)	1.00 (0.80)	0.85 (0.54)	<.001

ALB = albumin, ALT = alanine aminotransferase, AST = aspartate aminotransferase, BMI = body mass index, CVD = cardiovascular disease, FIB-4 = Fibrosis-4 index, HSI = hepatic steatosis index, NFS = nonalcoholic fatty liver disease fibrosis score, OA = osteoarthritis, PIR = poverty income ratio, PLT = platelet count, Q1–Q4 = quartiles 1–4, SD = standard deviation.

* Mean (SD); n (unweighted; %).

† Design-based Kruskal–Wallis test; Pearson *χ*^2^: Rao & Scott adjustment.

‡ Overall N refers to participants with non-missing HSI and valid HSI quartile assignment.

Across HSI quartiles, mean BMI increased from 21.90 kg/m^2^ in Q1 to 36.87 kg/m^2^ in Q4, and the prevalence of diabetes increased from 1.5% to 16% (both *P* < .001). Because BMI and diabetes are explicit components of the HSI formula, these gradients are expected by construction and should not be interpreted as independent clinical associations. Hypertension prevalence also increased across quartiles (14%–39%, *P* < .001). The prevalence of OA rose in parallel, doubling from 7.5% in Q1 to 16% in Q4 (*P* < .001). By contrast, age varied only modestly across HSI quartiles (mean range: 42.24–46.74 years), suggesting that HSI, as designed, differentiates participants more by steatosis-related metabolic features than by chronological age in this general population.

In contrast, both NFS and FIB-4 exhibited pronounced age gradients across quartiles (Tables S2 and S3). Mean age increased from 33.37 years in NFS Q1 to 61.72 years in Q4, and from 29.18 years in FIB-4 Q1 to 64.90 years in Q4 (both *P* < .001). For FIB-4, this age gradient is expected by construction because age is a direct component of the score formula and therefore should not be interpreted as an independent demographic pattern within the cohort. Consistent with the formulas and their clinical intent, higher quartiles were also characterized by lower platelet counts and lower albumin levels, along with a higher burden of comorbidities (e.g., hypertension and CVD history). OA prevalence increased sharply across both scores, from 3.4% to 29% across NFS quartiles and from 2.6% to 27% across FIB-4 quartiles (all *P* < .001). Notably, BMI increased across NFS quartiles (25.49–32.58 kg/m^2^), whereas BMI did not increase with FIB-4 severity and was slightly lower in Q4 (27.96 kg/m^2^) than in Q1 (28.86 kg/m^2^). Overall, these patterns suggest that NFS and especially FIB-4 are driven more by age- and fibrosis-related score components, whereas HSI better reflects a steatosis-oriented metabolic profile; these gradients should therefore be interpreted in light of the score formulas rather than as independent cohort-derived phenotypes.

### 3.2. Association between liver scores and OA

Because OA was a common outcome in this population (weighted prevalence, 12%), we prespecified survey-weighted modified Poisson regression with robust variance as the primary analytic approach to estimate PRs, whereas survey-weighted logistic regression was performed only as a secondary analysis and is presented in the Supplemental Digital Content.

In the primary Poisson models (Table 2), HSI showed the strongest and most consistent association with prevalent OA across all adjustment levels. In the crude model, participants in the highest HSI quartile had a higher OA prevalence than those in the lowest quartile (Q4 vs Q1: PR = 2.07, 95% CI = 1.86–2.31; *P* < .001). This association remained robust after full multivariable adjustment (Model 3: PR = 2.09, 95% CI = 1.87–2.33; *P* < .001). When modeled continuously, HSI also showed a clear dose–response relationship (per 1-SD increment: Model 3: PR = 1.34, 95% CI = 1.29–1.39; *P* < .001; *P* for trend < .001).

**Table 2:** Poisson regression (liver scores).

Exposure	Level	PR (95% CI)	*P* value	PR (95% CI)	*P* value	PR (95% CI)	*P* value	PR (95% CI)	*P* value
HSI	Per 1-SD increment	1.258 (1.214–1.304)	<.001	1.354 (1.294–1.416)	<.001	1.388 (1.342–1.435)	<.001	1.336 (1.289–1.385)	<.001
	Q1 (Ref)	1.00 (Reference)		1.00 (Reference)		1.00 (Reference)		1.00 (Reference)	
	Q2	1.487 (1.329–1.662)	<.001	1.321 (1.194–1.462)	<.001	1.317 (1.176–1.474)	<.001	1.282 (1.144–1.436)	<.001
	Q3	1.706 (1.513–1.924)	<.001	1.611 (1.453–1.785)	<.001	1.611 (1.433–1.810)	<.001	1.522 (1.353–1.713)	<.001
	Q4	2.073 (1.858–2.312)	<.001	2.311 (2.098–2.546)	<.001	2.324 (2.086–2.589)	<.001	2.088 (1.869–2.331)	<.001
	*P* for trend		<.001		<.001		<.001		<.001
NFS	Per 1-SD increment	1.952 (1.903–2.002)	<.001	1.249 (1.196–1.304)	<.001	1.247 (1.190–1.307)	<.001	1.190 (1.135–1.246)	<.001
	Q1 (Ref)	1.00 (Reference)		1.00 (Reference)		1.00 (Reference)		1.00 (Reference)	
	Q2	1.880 (1.597–2.212)	<.001	1.320 (1.128–1.545)	<.001	1.267 (1.076–1.491)	.005	1.248 (1.062–1.467)	.008
	Q3	3.997 (3.484–4.585)	<.001	1.713 (1.469–1.999)	<.001	1.628 (1.386–1.913)	<.001	1.586 (1.350–1.863)	<.001
	Q4	8.473 (7.452–9.634)	<.001	2.100 (1.792–2.460)	<.001	1.994 (1.693–2.348)	<.001	1.821 (1.547–2.143)	<.001
	*P* for trend		<.001		<.001		<.001		<.001
FIB-4	Per 1-SD increment	1.137 (1.103–1.173)	<.001	0.944 (0.898–0.992)	.024	0.947 (0.897–1.001)	.053	0.974 (0.927–1.024)	.306
	Q1 (Ref)	1.00 (Reference)		1.00 (Reference)		1.00 (Reference)		1.00 (Reference)	
	Q2	2.423 (2.031–2.890)	<.001	1.275 (1.065–1.525)	.008	1.221 (1.008–1.480)	.041	1.230 (1.019–1.485)	.031
	Q3	5.882 (4.937–7.007)	<.001	1.580 (1.296–1.927)	<.001	1.505 (1.220–1.858)	<.001	1.558 (1.270–1.912)	<.001
	Q4	10.325 (8.761–12.167)	<.001	1.337 (1.066–1.678)	.012	1.294 (1.018–1.643)	.035	1.423 (1.131–1.790)	.003
	*P* for trend		<.001		.158		.198		.011

Models: crude (unadjusted); Model 1 (age, sex, race); Model 2 (+SES, alcohol); Model 3 (+Hypertension, CVD). BMI and diabetes are adjusted only for FIB-4 (as they are components of HSI/NFS).

BMI = body mass index, CI = confidence interval, CVD = cardiovascular disease, FIB-4 = Fibrosis-4 index, HSI = hepatic steatosis index, NFS = nonalcoholic fatty liver disease fibrosis score, PR = prevalence ratio, Q1–Q4 = quartiles 1–4, Ref = reference, SD = standard deviation, SES = socioeconomic status.

NFS was also positively associated with OA, although the magnitude of association was substantially attenuated after adjustment, particularly after accounting for demographic factors. The crude association was strong (Q4 vs Q1: PR = 8.47, 95% CI = 7.45–9.63; *P* < .001), but decreased markedly in Model 1 (PR = 2.10, 95% CI = 1.79–2.46; *P* < .001) and remained significant in the fully adjusted model (Model 3: PR = 1.82, 95% CI = 1.55–2.14; *P* < .001). The continuous association was likewise significant (per 1-SD increment: Model 3: PR = 1.19, 95% CI = 1.14–1.25; *P* < .001), indicating an intermediate pattern between HSI and FIB-4.

By contrast, FIB-4 showed substantial attenuation after demographic adjustment, suggesting that much of its crude association with OA reflected age-related confounding. Although the crude association was strong (Q4 vs Q1: PR = 10.33, 95% CI = 8.76–12.17; *P* < .001), it was markedly reduced in Model 1 (PR = 1.34, 95% CI = 1.07–1.68; *P* = .012). In the fully adjusted model incorporating BMI and diabetes, a modest association persisted for the highest quartile (Model 3: Q4 vs Q1: PR = 1.42, 95% CI = 1.13–1.79; *P* = .003), whereas the continuous association remained null (per 1-SD increment: PR = 0.97, 95% CI = 0.93–1.02; *P* = .306).

Overall, under the predefined score-specific adjustment strategy, Table 2 shows a clear hierarchical pattern across the 3 indices: HSI had the strongest and most stable adjusted association with OA, NFS showed an intermediate association, and FIB-4 showed the weakest and least consistent adjusted signal. Secondary survey-weighted logistic regression analyses yielded directionally consistent results (Table S4), supporting that the main comparative conclusion was not dependent on the specific regression scale.

### 3.3. Dose–response relationships from restricted cubic spline analyses

**Figure 2.**
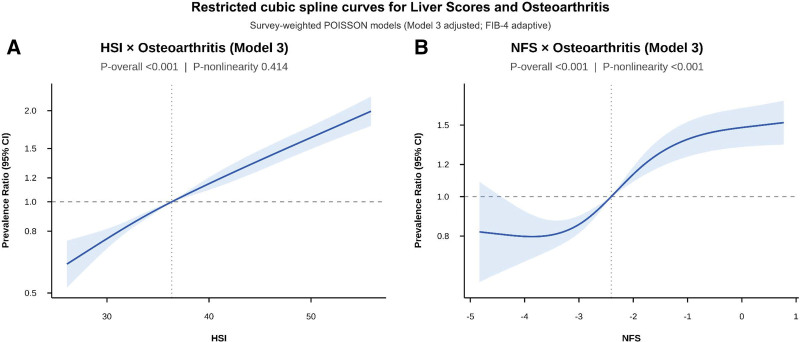
Restricted cubic spline curves showing the dose–response associations of HSI (A) and NFS (B) with osteoarthritis prevalence based on survey-weighted Poisson models (Model 3). FIB-4 = Fibrosis-4 index, HSI = hepatic steatosis index, NFS = NAFLD fibrosis score.

Restricted cubic spline analyses in fully adjusted Poisson models (Model 3) showed distinct dose–response patterns across the 3 indices (Figs. 2 and S1): HSI increased steadily, NFS showed a threshold-like pattern, and FIB-4 was less stable with wider uncertainty at the extremes. For HSI, OA prevalence increased monotonically across the exposure distribution without evidence of significant nonlinearity (*P* for overall association < .001; *P* for nonlinearity = .414), consistent with an approximately log-linear dose–response relationship. NFS exhibited significant nonlinearity (*P* for overall association < .001; *P* for nonlinearity < .001), with a relatively flat association at lower values followed by a steeper gradient at higher NFS levels, suggesting a threshold effect. FIB-4 also showed marked nonlinearity (*P* for overall association < .001; *P* for nonlinearity < .001), with a steep initial rise in PRs at lower FIB-4 values that plateaued at higher levels, accompanied by widening CIs at distribution extremes – a pattern consistent with age-driven confounding rather than a true dose-dependent metabolic signal. These spline findings reinforce the primary comparative result from Table 2.

### 3.4. Subgroup and interaction analyses

**Figure 3.**
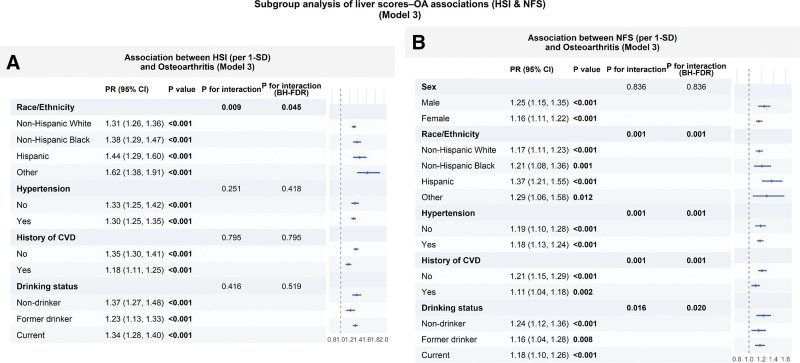
Subgroup analyses of the associations between HSI (A) and NFS (B) per 1-SD increase and osteoarthritis prevalence (PRs with 95% CIs) in the fully adjusted model, with interaction *P* values reported. CI = confidence interval, HSI = hepatic steatosis index, NFS = NAFLD fibrosis score, PRs = prevalence ratios, SD = standard deviation.

Prespecified subgroup analyses using fully adjusted survey-weighted Poisson models (Model 3) and per-SD liver scores showed a consistent pattern (Figs. 3 and S2): HSI and NFS remained positively associated with OA across most strata, whereas FIB-4 stayed near the null despite some statistically significant interaction tests. Interaction *P* values were adjusted using the BH-FDR procedure.

For HSI (Fig. 3A), the association with OA was consistently positive across all examined strata, with per 1-SD PRs ranging from 1.23 to 1.62, and all stratum-specific estimates statistically significant. Modest effect modification by race/ethnicity was detected (*P* for interaction = .009; BH-FDR-adjusted *P* = .045), with stronger associations observed in the “Other” race category (PR = 1.62, 95% CI = 1.38–1.91) compared with non-Hispanic White participants (PR = 1.31, 95% CI = 1.26–1.36). In contrast, interactions by hypertension, history of CVD, and drinking status were not statistically significant after BH-FDR correction (all BH-FDR > 0.05), indicating broadly consistent associations across these subgroups.

For NFS (Fig. 3B), per 1-SD increases were also associated with higher OA prevalence in all strata (PRs = 1.11–1.37). Unlike HSI, several interaction tests remained significant after BH-FDR adjustment, including race/ethnicity (BH-FDR = 0.001), hypertension (BH-FDR = 0.001), history of CVD (BH-FDR = 0.001), and drinking status (BH-FDR = 0.020). Race/ethnicity-stratified estimates suggested a comparatively stronger association among Hispanic participants (PR = 1.37, 95% CI = 1.21–1.55) than among non-Hispanic White participants (PR = 1.17, 95% CI = 1.11–1.23). The association appeared attenuated among those with a history of CVD (PR = 1.11, 95% CI = 1.04–1.18) compared with those without CVD (PR = 1.21, 95% CI = 1.15–1.29). No evidence of interaction by sex was observed (BH-FDR = 0.836).

For FIB-4 (Fig. S2,), stratum-specific associations were uniformly close to the null (PRs = 0.94–1.00, approximately), and none of the subgroup-specific estimates indicated a clear positive association. Although interaction tests for race/ethnicity, diabetes, hypertension, and history of CVD reached statistical significance after BH-FDR correction, the absolute differences between strata were small and the CIs overlapped substantially. Clinically, these findings suggest limited heterogeneity around an overall weak association rather than a robust subgroup-specific risk signal. Differences across race/ethnicity and cardiometabolic strata may reflect variations in age structure, aminotransferase patterns, platelet levels, comorbidity burden, score calibration, and background OA risk; therefore, these interactions do not support FIB-4 as a clinically actionable tool for identifying OA-prone subgroups.

### 3.5. Sensitivity analysis

**Figure 4.**
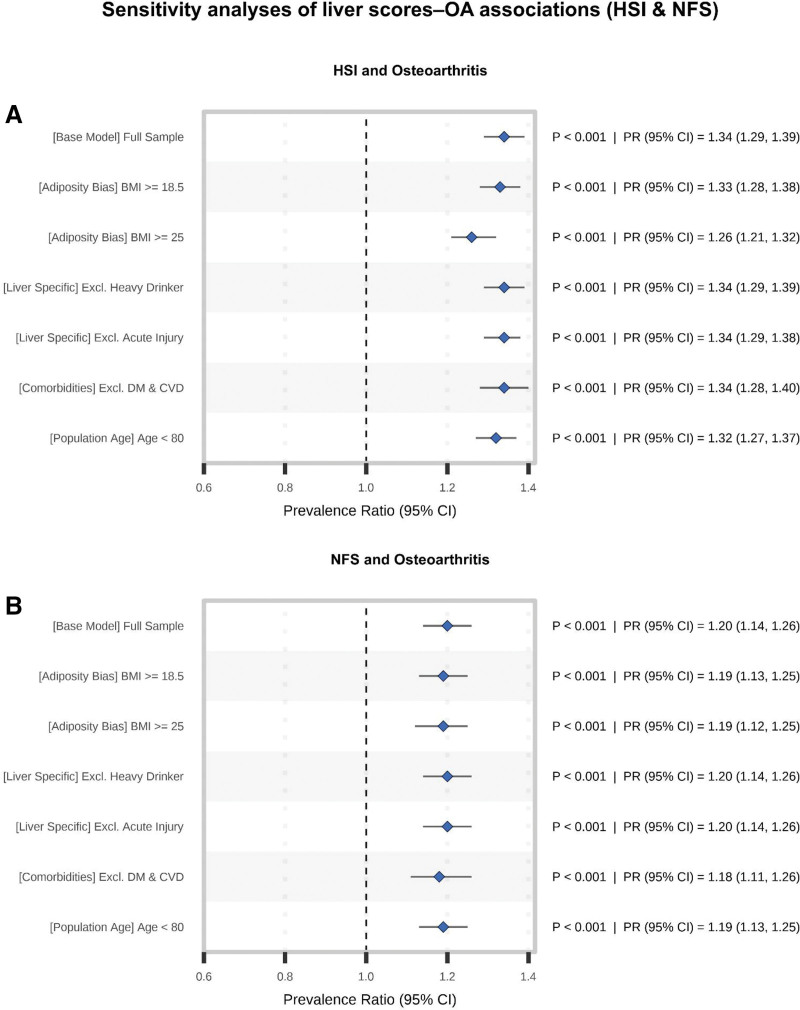
Sensitivity analyses for HSI (A) and NFS (B) showing consistent associations with OA prevalence (PRs with 95% CIs) across alternative sample restrictions, based on survey-weighted Poisson regression models (Model 3). CI = confidence interval, HSI = hepatic steatosis index, NFS = NAFLD fibrosis score, OA = osteoarthritis, PRs = prevalence ratios.

Sensitivity analyses confirmed the robustness of the primary findings (Figs. 4 and S3): HSI and NFS remained positively associated with OA across alternative restrictions, whereas FIB-4 stayed near the null in most scenarios. In Figure 4A, B, HSI and NFS showed little material change across prespecified restrictions related to adiposity, alcohol exposure, possible acute liver injury, major comorbidities, and advanced age. For HSI, the full-sample per 1-SD association was PR 1.34 (95% CI = 1.29–1.39; *P* < .001), and all restricted-sample estimates remained directionally consistent and statistically significant. NFS showed the same pattern, with a full-sample per 1-SD PR of 1.19 (95% CI = 1.14–1.26; *P* < .001) and comparable estimates across restricted samples. In contrast, the sensitivity analysis for FIB-4 (Fig. S3,) showed PRs close to the null in the full sample (PR = 0.97, 95% CI = 0.92–1.02; *P* = .306) and across most restricted samples, with the only notable deviation observed after excluding potential acute liver injury (PR = 0.95, 95% CI = 0.92–0.99; *P* = .019), suggesting an overall weak and inconsistent association compared with HSI and NFS. Collectively, these robustness checks confirm that HSI and NFS – indices more directly capturing metabolic liver burden – demonstrate consistent positive associations with OA that are insensitive to alternative analytic specifications or sample restrictions.

## 4. Discussion

In this large, nationally representative analysis of US adults, we systematically compared 3 widely used noninvasive liver indices and observed a clear gradient in their adjusted associations with OA prevalence. This pattern is broadly consistent with prior epidemiologic evidence linking fatty liver disease to OA. In a Korean KNHANES cross-sectional study, MAFLD defined by the fatty liver index was associated with radiographic knee OA.^[24]^ In NHANES 2017 to 2018, Lu et al reported that vibration-controlled transient elastography-defined NAFLD was significantly associated with self-reported OA after multivariable adjustment.^[25]^ A recent UK Biobank prospective cohort study further suggested that MAFLD was associated with a higher risk of incident knee OA.^[26]^ Against this background, our study extends the existing literature by directly comparing HSI, NFS, and FIB-4 within a unified analytic framework and showing that steatosis-oriented indices – particularly HSI – display stronger and more consistent adjusted associations with OA than the more age-dependent fibrosis marker FIB-4. Differences in effect size across studies may reflect variation in study population, study design, exposure definition, and OA ascertainment. Taken together, these findings support the view that steatosis-related metabolic dysfunction may be a more relevant hepatic correlate of OA in the general population than fibrosis burden alone.

### 4.1. Metabolic burden as a plausible “common soil” for liver-joint comorbidity

OA is increasingly recognized as a heterogeneous whole-joint disorder in which mechanical loading interacts with inflammatory and metabolic pathways.^[4,6]^ Under the “metabolic phenotype” framework, cardiometabolic abnormalities contribute to synovial inflammation, altered cartilage homeostasis, and subchondral bone remodeling through endocrine and immune mechanisms rather than biomechanics alone. In this context, it is biologically plausible that HSI – which directly incorporates markers of adiposity, diabetes, and aminotransferase patterns reflecting steatotic burden – would demonstrate the strongest adjusted association with OA. Importantly, the persistence of the HSI association after score-specific multivariable adjustment argues against it being explained solely by sociodemographic and lifestyle confounding; however, because HSI embeds BMI and diabetes status, the association should be interpreted as reflecting a steatosis-oriented metabolic phenotype rather than a fully independent effect of hepatic steatosis itself.

Mechanistically, hepatic steatosis and dysfunctional adipose tissue act as sources of chronic low-grade systemic inflammation and metabolic stress.^[10,11]^ This pro-inflammatory milieu is characterized by dysregulated production of pro-inflammatory cytokines, adipokines, and hepatokines, coupled with insulin resistance and lipotoxicity – pathways that may adversely affect chondrocyte metabolism, extracellular matrix homeostasis, and synovial inflammation. While our cross-sectional design cannot establish directionality, the observed hierarchy across indices is consistent with steatosis/metabolic load serving as an important context linking hepatic and articular health, but it does not establish a causal liver-specific pathway.

### 4.2. Why fibrosis-centric indices may underperform for extrahepatic phenotypes in the general population

A central observation was the substantial attenuation of the FIB-4 association following demographic adjustment, particularly for age, together with its comparatively modest and inconsistent estimates in the fully adjusted model. This pattern is not unexpected when FIB-4 – a tool designed for hepatic fibrosis screening – is applied to a general population for an extrahepatic outcome. FIB-4 includes age as a major component, and both OA prevalence and many chronic conditions rise sharply with age; thus, crude associations can be inflated by shared age structure rather than by a disease-specific biological connection. In addition, prior studies and clinical guidance indicate that FIB-4 is less reliable at age extremes and should be interpreted cautiously when aminotransferase abnormalities may be driven by non-fibrotic or transient causes.^[32,33]^ From this perspective, the weak and unstable FIB-4-OA association likely reflects a fundamental “measurement-target mismatch”: FIB-4 was engineered to detect hepatic fibrosis risk – not the broader metabolic dysregulation hypothesized to drive OA pathogenesis.

### 4.3. NFS as an intermediate phenotype marker: partial preservation via metabolic components

The NFS occupies a conceptual middle ground. Like FIB-4, it incorporates age and therefore, is susceptible to age-related confounding in unadjusted analyses. However, NFS also embeds metabolic and nutritional/inflammatory correlates (including adiposity and dysglycemia-related terms, albumin, and platelet-related information), which may allow it to retain an independent association with OA even after demographic and lifestyle adjustment. Our findings align with this conceptual framework: while NFS underperformed relative to HSI, it retained a significant adjusted association in fully adjusted models, suggesting that inclusion of metabolic components may enhance the relevance of fibrosis-oriented indices to extrahepatic metabolic phenotypes such as OA.

### 4.4. Dose–response patterns, effect heterogeneity, and robustness

Restricted cubic spline analyses provided additional insights into heterogeneous dose–response patterns across indices. The relationship between HSI and OA appeared broadly monotonic without strong evidence of nonlinearity, consistent with a graded metabolic burden model. In contrast, NFS and FIB-4 displayed more apparent nonlinear patterns, which may reflect threshold effects, competing influences of age versus metabolic context, or reduced interpretability at the tails of these distributions in a general-population setting.

Subgroup analyses provide additional nuance. For HSI and NFS, the direction of association was generally consistent across major strata, with only modest evidence of effect modification in selected subgroups. For FIB-4, statistically significant interactions emerged across race/ethnicity and cardiometabolic subgroups such as diabetes, hypertension, and history of CVD, yet the stratum-specific estimates remained close to the null and largely overlapping. This pattern suggests that the observed heterogeneity is more likely to reflect differences in age structure, platelet counts, aminotransferase profiles, comorbidity structure, and background OA risk across subgroups than a clinically meaningful subgroup-specific fibrosis signal. In practical terms, FIB-4 may therefore be less suitable for subgroup-based OA risk stratification, particularly in populations where cardiometabolic comorbidities dominate the clinical phenotype and may overwhelm any weak fibrosis-related signal. Importantly, sensitivity analyses using survey-weighted Poisson models produced PR estimates that were directionally concordant with logistic regression results, reinforcing that the primary conclusions are not driven by the OR scale in a common outcome setting.

### 4.5. Clinical and research implications

These findings suggest that simple, routinely available indices that reflect steatosis-prone metabolic burden may be informative beyond liver-focused outcomes. Clinically, elevated HSI may help flag individuals whose OA burden coexists with a broader cardiometabolic-inflammatory profile, supporting integrated evaluation of metabolic and musculoskeletal health rather than a liver-specific interpretation. At the research level, the observed hierarchy across scores offers a pragmatic hypothesis: in population OA, steatosis/metabolic dysregulation may be more salient than fibrosis staging as captured by FIB-4. Correspondingly, the subgroup interaction pattern of FIB-4 does not support its use as a clinically actionable tool for identifying OA-prone demographic or cardiometabolic subgroups, because statistically significant heterogeneity was observed around an overall near-null association. Future longitudinal studies should evaluate whether reductions in HSI (reflecting metabolic improvement) associate with decreased OA incidence or slowed progression, and mechanistic investigations should interrogate candidate mediators – including hepatokines, adipokines, insulin resistance biomarkers, and systemic inflammatory profiles – to elucidate causal pathways within the liver-joint axis.

### 4.6. Strengths and limitations

Strengths of this study include the large, nationally representative sample, the use of complex survey methods to generate population-relevant inference, the head-to-head comparison of multiple indices within a harmonized analytic framework, and the consistent findings across primary Poisson and secondary logistic regression models, dose–response splines, and multiple sensitivity analyses. Several limitations merit emphasis. First, the cross-sectional design precludes causal inference and cannot exclude reverse causation (i.e., OA leading to reduced physical activity and subsequent metabolic deterioration). Second, OA ascertainment relied on self-reported physician diagnosis, which may introduce non-differential misclassification, likely attenuating observed associations toward the null. Third, noninvasive liver indices are imperfect surrogates for hepatic histology and imaging; because their components inherently overlap with established OA risk factors (e.g., BMI, diabetes), disentangling independent hepatic contributions from general metabolic burden remains challenging. Finally, residual confounding is possible despite extensive adjustment.

## 5. Conclusion

In this population-based analysis, noninvasive liver scores were differentially associated with OA. HSI showed the strongest and most consistent associations with OA, NFS showed intermediate performance, and FIB-4 exhibited weak and unstable associations largely influenced by age-related score structure. These findings suggest that steatosis-related metabolic dysregulation may be more relevant to OA burden than fibrosis burden alone in the general population. Because HSI incorporates BMI and diabetes status, its association with OA should be interpreted as that of a composite steatosis-oriented metabolic marker rather than a fully independent liver-specific effect. Future longitudinal and mechanistic research is warranted to establish causality and elucidate biological pathways linking liver metabolic health to joint disease.

## Author contributions

**Conceptualization:** Di Wang, Xin Zhao.

**Data curation:** Di Wang.

**Formal analysis:** Di Wang, Xin Zhao.

**Writing – original draft:** Di Wang.

**Validation:** Xin Zhao.

**Writing – review & editing:** Xin Zhao.














