# Supplementary material for: Metabolic liver burden and osteoarthritis prevalence: A comparative analysis of noninvasive hepatic indices
Source: Medicine (Baltimore). 2026 May 22;105(21):e48764. doi: 10.1097/MD.0000000000048764 (PMC13200982; doi:10.1097/MD.0000000000048764)
Supplement: Supplementary file 1 [file medi-105-e48764-s001.docx]

Supplementary file 1 Table S1. Baseline characteristics of the common analytic cohort (Weighted)

| **Characteristic** | **Overall** N = 40,447^1^ |
| --- | --- |
| **age** | 45.10 (16.56) |
| **sex** |  |
| Male | 19,898 (49%) |
| Female | 20,549 (51%) |
| **race** |  |
| Non-Hispanic White | 17,725 (68%) |
| Non-Hispanic Black | 7,860 (10%) |
| Hispanic | 10,819 (15%) |
| Other | 4,043 (7.2%) |
| **education** |  |
| >High school | 21,071 (61%) |
| High school | 9,169 (23%) |
| <High school | 10,159 (16%) |
| **PIR** | 3.04 (1.64) |
| **marital** |  |
| Married/Living with partner | 24,627 (64%) |
| Not married | 15,433 (36%) |
| **BMI** | 28.48 (6.58) |
| **drinking_status** |  |
| Never | 7,056 (15%) |
| Former | 4,089 (9.3%) |
| Current | 25,345 (75%) |
| **diabetes** | 4,021 (7.5%) |
| **hypertension** | 12,123 (27%) |
| **CVD_history** | 3,455 (6.8%) |
| **OA_case** | 4,666 (12%) |
| **ALT** | 25.53 (22.52) |
| **AST** | 25.07 (16.09) |
| **ALB** | 4.29 (0.35) |
| **PLT** | 253.58 (64.91) |
| **HSI** | 37.60 (7.77) |
| **NFS** | -2.30 (1.42) |
| **FIB4** | 1.00 (0.76) |
| ^1^Mean (SD); n (unweighted) (%) | |
| Note: The common analytic cohort included participants with at least one calculable liver score (HSI, NFS, or FIB-4). | |
| Abbreviations: OA, osteoarthritis; PIR, poverty income ratio; BMI, body mass index; CVD, cardiovascular disease; ALT, alanine aminotransferase; AST, aspartate aminotransferase; ALB, albumin; PLT, platelet count; HSI, hepatic steatosis index; NFS, nonalcoholic fatty liver disease fibrosis score; FIB-4, fibrosis-4 index; SD, standard deviation. | |
