# Supplementary material for: Metabolic liver burden and osteoarthritis prevalence: A comparative analysis of noninvasive hepatic indices
Source: Medicine (Baltimore). 2026 May 22;105(21):e48764. doi: 10.1097/MD.0000000000048764 (PMC13200982; doi:10.1097/MD.0000000000048764)
Supplement: Supplementary file 2 [file medi-105-e48764-s002.docx]

Supplementary file 2 Table S2. Characteristics by NFS Quartiles (Weighted)

| **Characteristic** | **Overall^3^** N = 39042^1^ | **Q1** N = 9761^1^ | **Q2** N = 9760^1^ | **Q3** N = 9760^1^ | **Q4** N = 9761^1^ | **P-value**^2^ |
| --- | --- | --- | --- | --- | --- | --- |
| **age** | 44.87 (16.48) | 33.37 (10.95) | 40.11 (12.50) | 49.52 (13.99) | 61.72 (14.39) | <0.001 |
| **sex** |  |  |  |  |  | 0.090 |
| Male | 19,151 (49%) | 4,760 (50%) | 4,686 (49%) | 4,652 (48%) | 5,053 (50%) |  |
| Female | 19,891 (51%) | 5,001 (50%) | 5,074 (51%) | 5,108 (52%) | 4,708 (50%) |  |
| **race** |  |  |  |  |  | <0.001 |
| Non-Hispanic White | 17,103 (68%) | 4,115 (65%) | 4,075 (66%) | 4,220 (69%) | 4,693 (73%) |  |
| Non-Hispanic Black | 7,576 (10%) | 1,495 (8.6%) | 1,839 (10%) | 2,038 (11%) | 2,204 (12%) |  |
| Hispanic | 10,476 (15%) | 3,010 (18%) | 2,736 (16%) | 2,547 (13%) | 2,183 (9.8%) |  |
| Other | 3,887 (7.2%) | 1,141 (8.5%) | 1,110 (7.6%) | 955 (6.8%) | 681 (5.2%) |  |
| **education** |  |  |  |  |  | <0.001 |
| >High school | 20,412 (61%) | 5,233 (60%) | 5,330 (62%) | 5,236 (63%) | 4,613 (57%) |  |
| High school | 8,851 (23%) | 2,240 (24%) | 2,167 (23%) | 2,214 (23%) | 2,230 (24%) |  |
| <High school | 9,742 (16%) | 2,279 (16%) | 2,253 (15%) | 2,304 (14%) | 2,906 (19%) |  |
| **PIR** | 3.05 (1.64) | 2.87 (1.66) | 3.11 (1.64) | 3.22 (1.63) | 3.01 (1.60) | <0.001 |
| **marital** |  |  |  |  |  | <0.001 |
| Married/Living with partner | 23,838 (64%) | 5,348 (56%) | 6,162 (66%) | 6,459 (71%) | 5,869 (65%) |  |
| Not married | 14,828 (36%) | 4,296 (44%) | 3,480 (34%) | 3,218 (29%) | 3,834 (35%) |  |
| **BMI** | 28.43 (6.55) | 25.49 (4.82) | 27.44 (5.33) | 29.51 (6.08) | 32.58 (8.07) | <0.001 |
| **drinking_status** |  |  |  |  |  | <0.001 |
| Never | 6,809 (15%) | 1,434 (14%) | 1,475 (13%) | 1,750 (15%) | 2,150 (21%) |  |
| Former | 3,883 (9.1%) | 527 (5.3%) | 687 (7.5%) | 951 (9.3%) | 1,718 (16%) |  |
| Current | 24,663 (76%) | 6,826 (81%) | 6,604 (79%) | 6,184 (75%) | 5,049 (63%) |  |
| **diabetes** | 3,908 (7.4%) | 60 (0.5%) | 190 (1.5%) | 620 (4.8%) | 3,038 (28%) | <0.001 |
| **hypertension** | 11,430 (26%) | 1,161 (12%) | 1,841 (18%) | 3,071 (30%) | 5,357 (53%) | <0.001 |
| **CVD_history** | 3,189 (6.5%) | 150 (1.4%) | 314 (3.0%) | 659 (5.7%) | 2,066 (20%) | <0.001 |
| **OA_case** | 4,381 (12%) | 297 (3.4%) | 543 (6.4%) | 1,135 (14%) | 2,406 (29%) | <0.001 |
| **ALT** | 25.54 (22.72) | 26.87 (29.97) | 25.54 (20.91) | 25.21 (17.42) | 24.08 (18.47) | <0.001 |
| **AST** | 25.09 (16.22) | 24.24 (13.80) | 24.49 (14.46) | 25.35 (15.98) | 26.76 (21.04) | <0.001 |
| **ALB** | 4.29 (0.35) | 4.44 (0.33) | 4.34 (0.31) | 4.23 (0.32) | 4.10 (0.34) | <0.001 |
| **PLT** | 253.63 (64.75) | 303.76 (65.63) | 253.70 (49.55) | 233.79 (48.22) | 207.42 (50.16) | <0.001 |
| **HSI** | 37.59 (7.77) | 35.01 (6.46) | 36.59 (6.76) | 38.51 (7.41) | 41.45 (9.29) | <0.001 |
| **NFS** | -2.30 (1.42) | -3.90 (0.66) | -2.68 (0.26) | -1.74 (0.30) | -0.22 (0.84) | <0.001 |
| **FIB4** | 1.00 (0.76) | 0.54 (0.19) | 0.79 (0.27) | 1.11 (0.42) | 1.78 (1.22) | <0.001 |
| ^1^Mean (SD); n (unweighted) (%) | | | | | | |
| ^2^Design-based KruskalWallis test; Pearson's X^2: Rao & Scott adjustment | | | | | | |
| ^3^Overall N refers to participants with non-missing NFS and valid NFS quartile assignment. | | | | | | |
| Abbreviations: Q1–Q4, quartiles 1–4; OA, osteoarthritis; PIR, poverty income ratio; BMI, body mass index; CVD, cardiovascular disease; ALT, alanine aminotransferase; AST, aspartate aminotransferase; ALB, albumin; PLT, platelet count; HSI, hepatic steatosis index; NFS, nonalcoholic fatty liver disease fibrosis score; FIB-4, fibrosis-4 index; SD, standard deviation. | | | | | | |
