# Supplementary material for: Metabolic liver burden and osteoarthritis prevalence: A comparative analysis of noninvasive hepatic indices
Source: Medicine (Baltimore). 2026 May 22;105(21):e48764. doi: 10.1097/MD.0000000000048764 (PMC13200982; doi:10.1097/MD.0000000000048764)
Supplement: Supplementary file 3 [file medi-105-e48764-s003.docx]

Supplementary file 3 Table S3. Characteristics by FIB-4 Quartiles (Weighted)

| **Characteristic** | **Overall^3^** N = 40380^1^ | **Q1** N = 10095^1^ | **Q2** N = 10095^1^ | **Q3** N = 10095^1^ | **Q4** N = 10095^1^ | **P-value**^2^ |
| --- | --- | --- | --- | --- | --- | --- |
| **age** | 45.10 (16.56) | 29.18 (7.85) | 39.88 (10.29) | 51.18 (11.54) | 64.90 (12.01) | <0.001 |
| **sex** |  |  |  |  |  | <0.001 |
| Male | 19,862 (49%) | 4,117 (44%) | 4,832 (50%) | 5,130 (50%) | 5,783 (53%) |  |
| Female | 20,518 (51%) | 5,978 (56%) | 5,263 (50%) | 4,965 (50%) | 4,312 (47%) |  |
| **race** |  |  |  |  |  | <0.001 |
| Non-Hispanic White | 17,696 (68%) | 3,851 (60%) | 4,123 (65%) | 4,402 (71%) | 5,320 (78%) |  |
| Non-Hispanic Black | 7,850 (10%) | 2,004 (12%) | 1,982 (11%) | 1,979 (9.7%) | 1,885 (8.8%) |  |
| Hispanic | 10,799 (15%) | 3,249 (21%) | 2,902 (16%) | 2,644 (12%) | 2,004 (7.5%) |  |
| Other | 4,035 (7.2%) | 991 (7.7%) | 1,088 (7.6%) | 1,070 (7.2%) | 886 (6.1%) |  |
| **education** |  |  |  |  |  | <0.001 |
| >High school | 21,043 (61%) | 5,293 (58%) | 5,575 (63%) | 5,348 (63%) | 4,827 (58%) |  |
| High school | 9,156 (23%) | 2,463 (25%) | 2,196 (22%) | 2,200 (22%) | 2,297 (24%) |  |
| <High school | 10,134 (16%) | 2,331 (16%) | 2,314 (15%) | 2,539 (15%) | 2,950 (18%) |  |
| **PIR** | 3.04 (1.64) | 2.60 (1.63) | 3.10 (1.63) | 3.36 (1.59) | 3.15 (1.59) | <0.001 |
| **marital** |  |  |  |  |  | <0.001 |
| Married/Living with partner | 24,588 (64%) | 5,210 (52%) | 6,536 (68%) | 6,733 (71%) | 6,109 (66%) |  |
| Not married | 15,406 (36%) | 4,781 (48%) | 3,439 (32%) | 3,273 (29%) | 3,913 (34%) |  |
| **BMI** | 28.48 (6.57) | 28.86 (7.36) | 28.50 (6.53) | 28.47 (6.26) | 27.96 (5.87) | <0.001 |
| **drinking_status** |  |  |  |  |  | <0.001 |
| Never | 7,048 (15%) | 1,620 (15%) | 1,515 (13%) | 1,790 (15%) | 2,123 (19%) |  |
| Former | 4,081 (9.3%) | 532 (5.1%) | 781 (8.0%) | 1,135 (10%) | 1,633 (15%) |  |
| Current | 25,299 (75%) | 6,929 (80%) | 6,719 (79%) | 6,240 (75%) | 5,411 (66%) |  |
| **diabetes** | 4,016 (7.5%) | 337 (2.9%) | 673 (5.1%) | 1,224 (8.9%) | 1,782 (15%) | <0.001 |
| **hypertension** | 12,103 (27%) | 1,275 (13%) | 2,109 (20%) | 3,591 (33%) | 5,128 (47%) | <0.001 |
| **CVD_history** | 3,448 (6.8%) | 138 (1.2%) | 350 (3.2%) | 840 (6.8%) | 2,120 (19%) | <0.001 |
| **OA_case** | 4,661 (12%) | 231 (2.6%) | 579 (6.4%) | 1,425 (16%) | 2,426 (27%) | <0.001 |
| **ALT** | 25.53 (22.53) | 24.74 (26.53) | 25.29 (15.85) | 25.51 (16.43) | 26.86 (29.71) | <0.001 |
| **AST** | 25.07 (16.10) | 21.43 (6.85) | 23.51 (7.91) | 25.34 (9.95) | 31.46 (30.44) | <0.001 |
| **ALB** | 4.29 (0.35) | 4.31 (0.39) | 4.32 (0.35) | 4.29 (0.33) | 4.23 (0.32) | <0.001 |
| **PLT** | 253.58 (64.91) | 295.67 (66.92) | 262.45 (56.92) | 241.07 (49.51) | 203.31 (47.21) | <0.001 |
| **HSI** | 37.59 (7.77) | 38.79 (8.73) | 37.83 (7.73) | 37.40 (7.30) | 35.93 (6.66) | <0.001 |
| **NFS** | -2.30 (1.42) | -3.55 (1.03) | -2.68 (0.90) | -1.88 (0.92) | -0.66 (1.09) | <0.001 |
| **FIB4** | 1.00 (0.76) | 0.44 (0.09) | 0.72 (0.08) | 1.08 (0.13) | 2.01 (1.12) | <0.001 |
| ^1^Mean (SD); n (unweighted) (%) | | | | | | |
| ^2^Design-based KruskalWallis test; Pearson's X^2: Rao & Scott adjustment | | | | | | |
| ^3^Overall N refers to participants with non-missing FIB-4 and valid FIB-4 quartile assignment. | | | | | | |
| Abbreviations: Q1–Q4, quartiles 1–4; OA, osteoarthritis; PIR, poverty income ratio; BMI, body mass index; CVD, cardiovascular disease; ALT, alanine aminotransferase; AST, aspartate aminotransferase; ALB, albumin; PLT, platelet count; HSI, hepatic steatosis index; NFS, nonalcoholic fatty liver disease fibrosis score; FIB-4, fibrosis-4 index; SD, standard deviation. | | | | | | |
