# Supplementary material for: Metabolic liver burden and osteoarthritis prevalence: A comparative analysis of noninvasive hepatic indices
Source: Medicine (Baltimore). 2026 May 22;105(21):e48764. doi: 10.1097/MD.0000000000048764 (PMC13200982; doi:10.1097/MD.0000000000048764)
Supplement: Supplementary file 4 [file medi-105-e48764-s004.docx]

Supplementary file 4 Table S4. Logistic Regression (Liver Scores)

| **Exposure** | **Level** | **OR (95% CI)** | **P value** | **OR (95% CI)** | **P value** | **OR (95% CI)** | **P value** | **OR (95% CI)** | **P value** |
| --- | --- | --- | --- | --- | --- | --- | --- | --- | --- |
| HSI | Per 1-SD increment | 1.326 (1.272, 1.383) | <0.001 | 1.549 (1.476, 1.625) | <0.001 | 1.561 (1.480, 1.646) | <0.001 | 1.478 (1.399, 1.563) | <0.001 |
|  | Q1 (Ref) | 1.00 (Reference) |  | 1.00 (Reference) |  | 1.00 (Reference) |  | 1.00 (Reference) |  |
|  | Q2 | 1.548 (1.369, 1.751) | <0.001 | 1.432 (1.249, 1.643) | <0.001 | 1.435 (1.231, 1.673) | <0.001 | 1.379 (1.180, 1.612) | <0.001 |
|  | Q3 | 1.810 (1.584, 2.069) | <0.001 | 1.867 (1.622, 2.148) | <0.001 | 1.881 (1.601, 2.209) | <0.001 | 1.733 (1.472, 2.040) | <0.001 |
|  | Q4 | 2.272 (2.010, 2.567) | <0.001 | 3.011 (2.638, 3.437) | <0.001 | 3.065 (2.636, 3.564) | <0.001 | 2.638 (2.261, 3.077) | <0.001 |
|  | P for trend |  | <0.001 |  | <0.001 |  | <0.001 |  | <0.001 |
| NFS | Per 1-SD increment | 2.389 (2.291, 2.492) | <0.001 | 1.371 (1.287, 1.459) | <0.001 | 1.364 (1.272, 1.462) | <0.001 | 1.277 (1.191, 1.370) | <0.001 |
|  | Q1 (Ref) | 1.00 (Reference) |  | 1.00 (Reference) |  | 1.00 (Reference) |  | 1.00 (Reference) |  |
|  | Q2 | 1.940 (1.635, 2.301) | <0.001 | 1.269 (1.067, 1.510) | 0.007 | 1.222 (1.019, 1.464) | 0.031 | 1.200 (1.001, 1.439) | 0.049 |
|  | Q3 | 4.466 (3.858, 5.169) | <0.001 | 1.665 (1.400, 1.980) | <0.001 | 1.593 (1.325, 1.914) | <0.001 | 1.547 (1.285, 1.862) | <0.001 |
|  | Q4 | 11.477 (9.971, 13.209) | <0.001 | 2.338 (1.953, 2.799) | <0.001 | 2.226 (1.844, 2.688) | <0.001 | 1.966 (1.626, 2.377) | <0.001 |
|  | P for trend |  | <0.001 |  | <0.001 |  | <0.001 |  | <0.001 |
| FIB-4 | Per 1-SD increment | 1.751 (1.604, 1.911) | <0.001 | 0.935 (0.872, 1.003) | 0.060 | 0.938 (0.868, 1.013) | 0.100 | 0.974 (0.908, 1.044) | 0.455 |
|  | Q1 (Ref) | 1.00 (Reference) |  | 1.00 (Reference) |  | 1.00 (Reference) |  | 1.00 (Reference) |  |
|  | Q2 | 2.520 (2.098, 3.026) | <0.001 | 1.115 (0.920, 1.351) | 0.266 | 1.079 (0.877, 1.328) | 0.467 | 1.139 (0.924, 1.403) | 0.222 |
|  | Q3 | 6.779 (5.639, 8.151) | <0.001 | 1.359 (1.096, 1.684) | 0.005 | 1.313 (1.045, 1.649) | 0.020 | 1.485 (1.181, 1.869) | <0.001 |
|  | Q4 | 13.821 (11.616, 16.445) | <0.001 | 1.130 (0.873, 1.464) | 0.351 | 1.114 (0.846, 1.466) | 0.439 | 1.360 (1.033, 1.791) | 0.029 |
|  | P for trend |  | <0.001 |  | 0.555 |  | 0.557 |  | 0.027 |
| Abbreviations: OR, odds ratio; CI, confidence interval; SD, standard deviation; Ref, reference; Q1–Q4, quartiles 1–4; HSI, hepatic steatosis index; NFS, nonalcoholic fatty liver disease fibrosis score; FIB-4, fibrosis-4 index; SES, socioeconomic status; BMI, body mass index; CVD, cardiovascular disease. | | | | | | | | | |
| Models: Crude (unadjusted); Model 1 (age, sex, race); Model 2 (+SES, Alcohol). | | | | | | | | | |
| Model 3 (+Hypertension, CVD). | | | | | | | | | |
| Note: BMI and Diabetes are adjusted ONLY for FIB-4 (as they are components of HSI/NFS). | | | | | | | | | |
| Bold indicates P < 0.05. | | | | | | | | | |
