# Supplementary material for: Metabolic liver burden and osteoarthritis prevalence: A comparative analysis of noninvasive hepatic indices
Source: Medicine (Baltimore). 2026 May 22;105(21):e48764. doi: 10.1097/MD.0000000000048764 (PMC13200982; doi:10.1097/MD.0000000000048764)
Supplement: Supplementary file 5 [file medi-105-e48764-s005.docx]

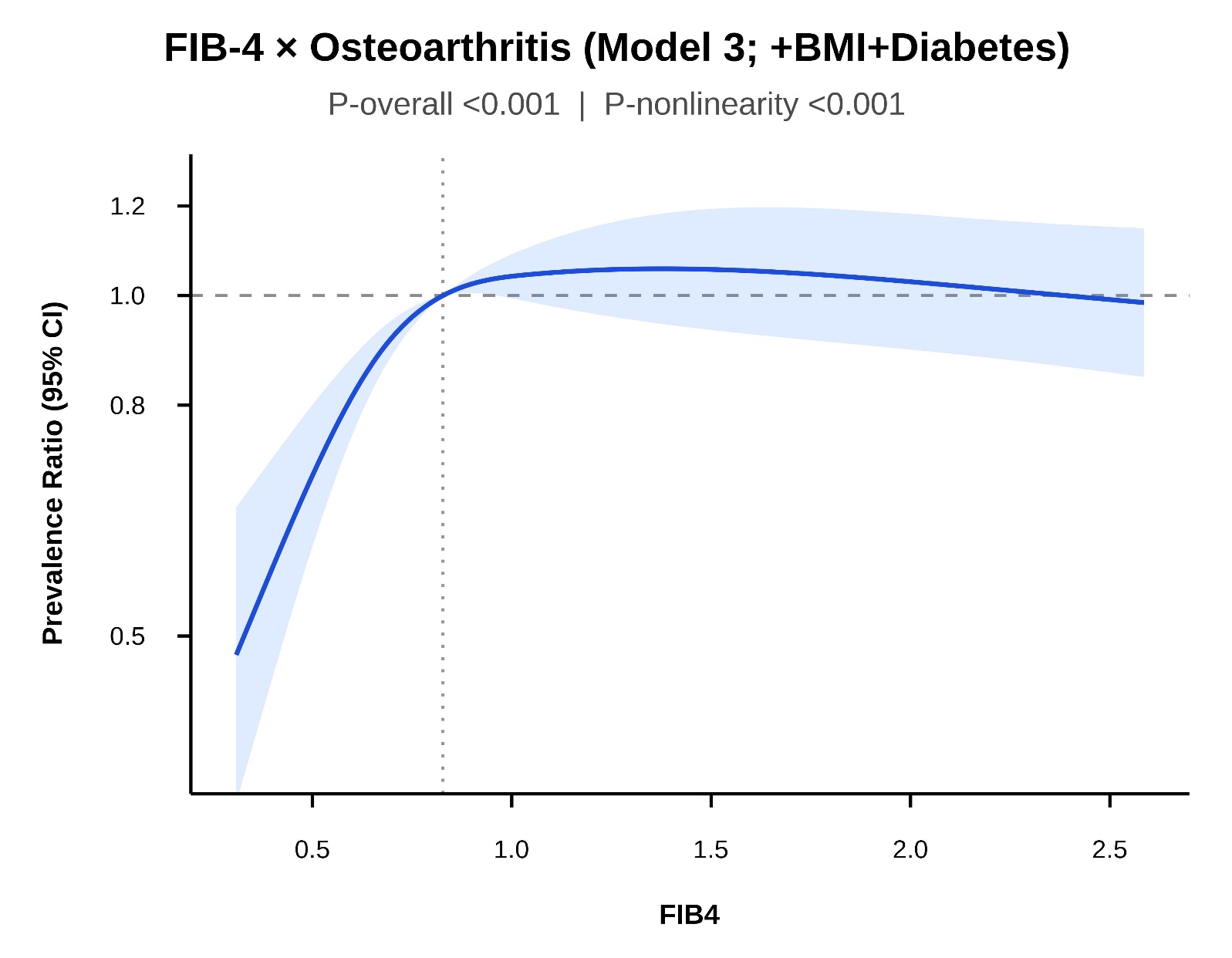


Supplementary file 5 Figure S1. Restricted cubic spline curve showing the dose–response association of FIB-4 with osteoarthritis prevalence based on the fully adjusted survey-weighted Poisson model (Model 3 with additional adjustment for BMI and diabetes).
