# Supplementary material for: Metabolic liver burden and osteoarthritis prevalence: A comparative analysis of noninvasive hepatic indices
Source: Medicine (Baltimore). 2026 May 22;105(21):e48764. doi: 10.1097/MD.0000000000048764 (PMC13200982; doi:10.1097/MD.0000000000048764)
Supplement: Supplementary file 6 [file medi-105-e48764-s006.docx]

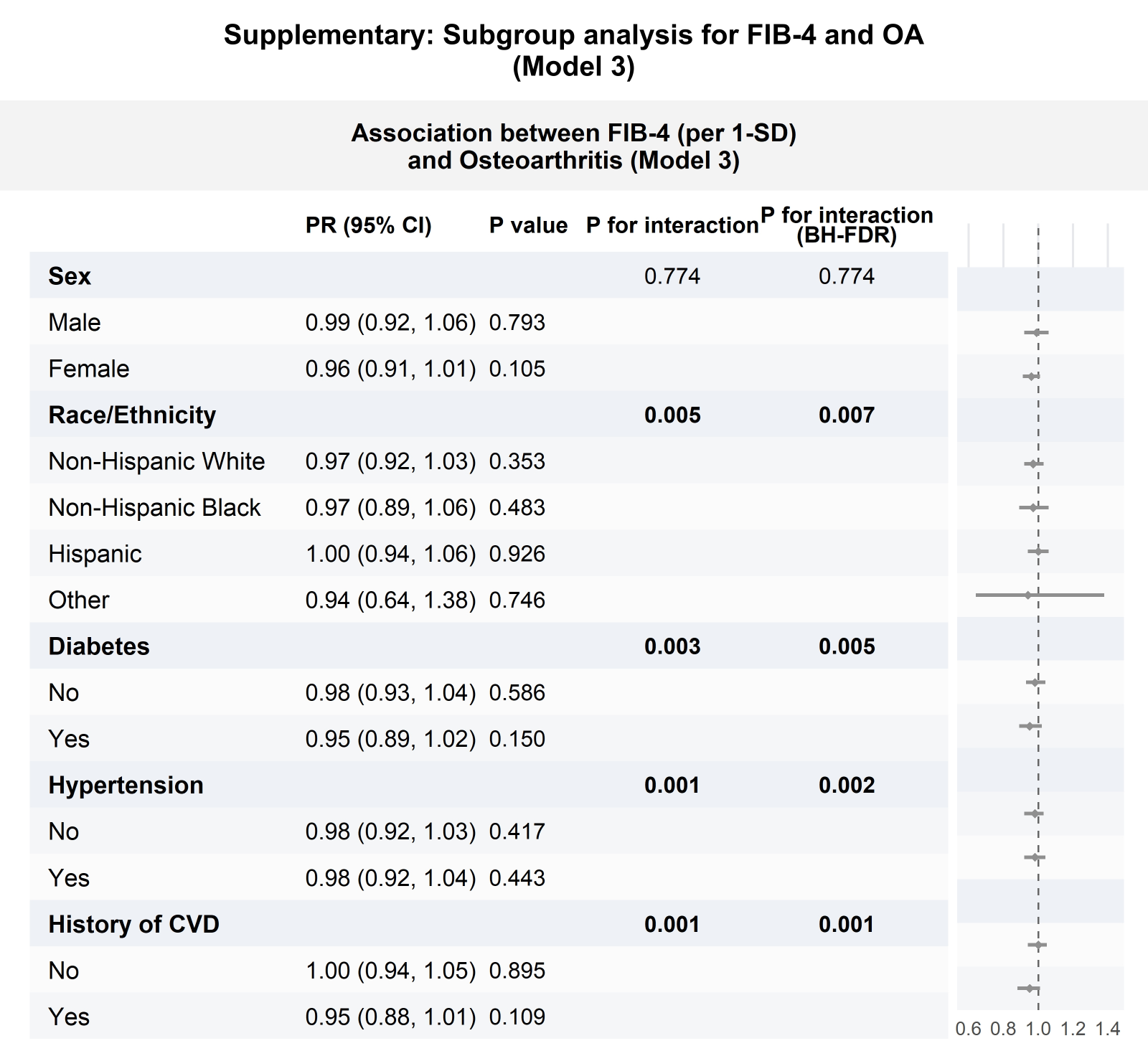


Supplementary file 6 Figure S2. Subgroup analyses of the association between FIB-4 per 1-SD increase and osteoarthritis prevalence (PRs with 95% CIs) in the fully adjusted model, with interaction P values reported.
