# Supplementary material for: Metabolic liver burden and osteoarthritis prevalence: A comparative analysis of noninvasive hepatic indices
Source: Medicine (Baltimore). 2026 May 22;105(21):e48764. doi: 10.1097/MD.0000000000048764 (PMC13200982; doi:10.1097/MD.0000000000048764)
Supplement: Supplementary file 7 [file medi-105-e48764-s007.docx]

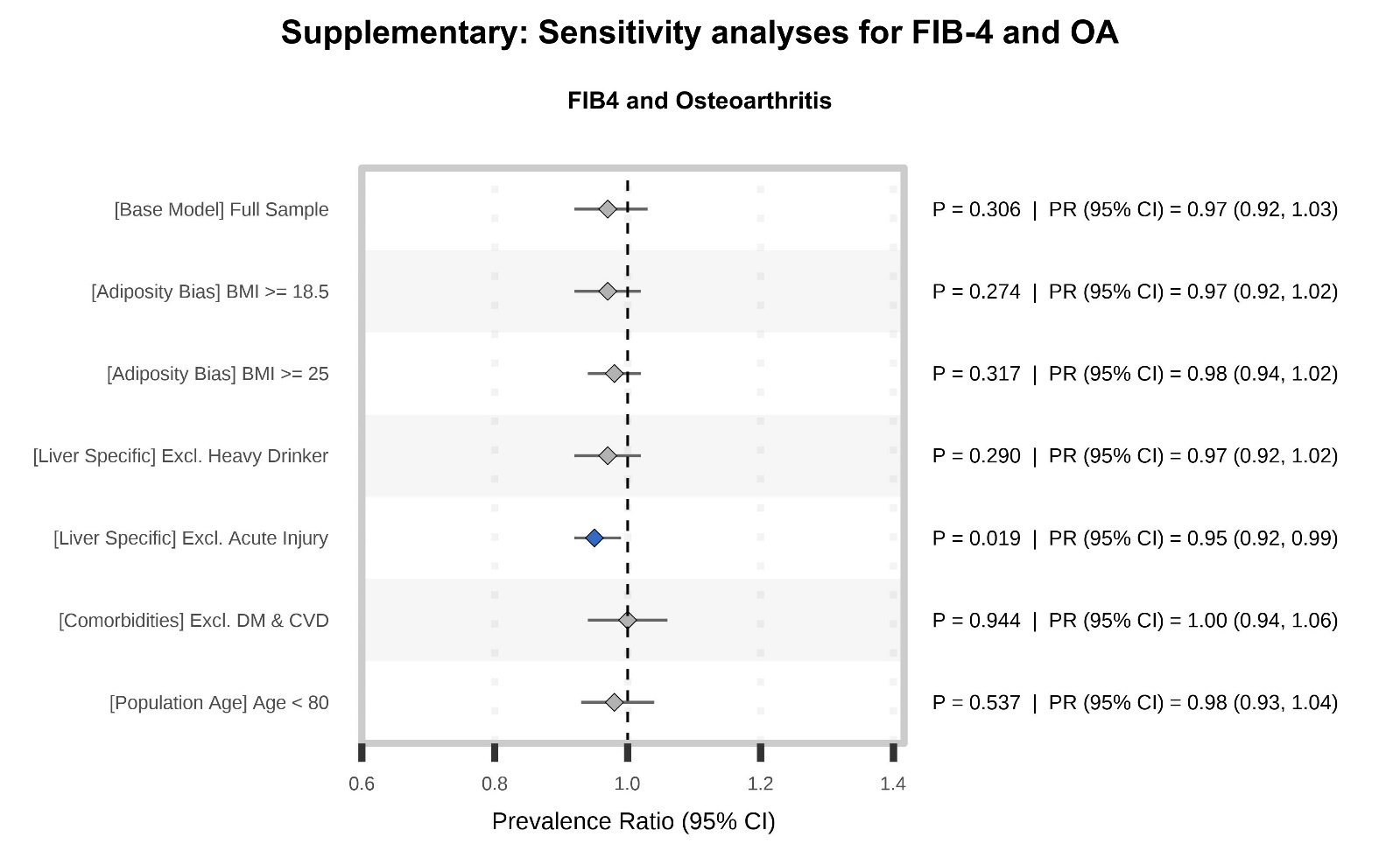


Supplementary file 7 Figure S3. Sensitivity analyses for FIB-4 showing the association with OA prevalence (PRs with 95% CIs) across alternative analytic restrictions in survey-weighted Poisson models.
